# Organic-Inorganic Hydrophobic Nanocomposite Film with a Core-Shell Structure

**DOI:** 10.3390/ma9121021

**Published:** 2016-12-17

**Authors:** Peng Liu, Ying Chen, Zhiwu Yu

**Affiliations:** 1 School of Civil Engineering, Central South University, 22 Shaoshan Road, Changsha 410075, China; lop868@163.com (P.L.); zhwyu@csu.edu.cn (Z.Y.); 2 School of Civil Engineering, Shenzhen University, 3688 Nanhai Road, Shenzhen 518060, China; 3 National Engineering Laboratory for High Speed Railway Construction, Central South University, Changsha 410075, China; 4 Graduate School at Shenzhen, Tsing Hua University, 2279 Lishui Road, Shenzhen 518055, China

**Keywords:** core-shell structure, ternary nanoparticles, crystal, monomer, emulsion

## Abstract

A method to prepare novel organic-inorganic hydrophobic nanocomposite films was proposed by a site-specific polymerization process. The inorganic part, the core of the nanocomposite, is a ternary SiO_2_–Al_2_O_3_–TiO_2_ nanoparticles, which is grafted with methacryloxy propyl trimethoxyl silane (KH570), and wrapped by fluoride and siloxane polymers. The synthesized samples are characterized by transmission electron microscopy (TEM), Fourier transform infrared (FTIR) spectrscopy, X-ray diffractometry (XRD), contact angle meter (CA), and scanning electron microscope (SEM). The results indicate that the novel organic-inorganic hydrophobic nanocomposite with a core-shell structure was synthesized successfully. XRD analysis reveals the nanocomposite film has an amorphous structure, and FTIR analysis indicates the nanoparticles react with a silane coupling agent (methacryloxy propyl trimethoxyl silane KH570). Interestingly, the morphology of the nanoparticle film is influenced by the composition of the core. Further, comparing with the film synthesized by silica nanoparticles, the film formed from SiO_2_–Al_2_O_3_–TiO_2_ nanoparticles has higher hydrophobic performance, i.e., the contact angle is greater than 101.7°. In addition, the TEM analysis reveals that the crystal structure of the particles can be changed at high temperatures.

## 1. Introduction

To address the issues of traditional polymer materials, such as low strength and poor thermal stability, different efforts have been made to develop polymer composites [1,2,3]. Explicitly, nanomaterials have several unique properties, such as quantum effect and specific surface effects. If the nanomaterials can be introduced into polymer, the prepared nanocomposite may have novel aging resistance, thermal stability, and so on, which has become a hot topic [4,5,6,7,8,9] in the fields of coating, catalysis, energy storage, and water treatment [10,11]. Cho et al. [12] presented new inorganic-organic hybrid particles using as an adsorbent for simultaneous removal of hydrophobic and hydrophilic pollutants from water. A kind of PANI/SiO_2_ nanocomposite emulsion was synthesized by using polyaniline and silica nanoparticles [13]. An emulsion polymerization method was adopted to prepare SiO_2_/P(MMA/BA/3FMA) organic-inorganic nanocomposite with a emulsion structure [14]. Moreover, various nanocomposite particles with a core/shell structure were fabricated and used in many engineering application [15,16,17].

At present, the existing achievements are mainly embodied in new product and preparation method [18]. Numerous new nanoparticles and nanocomposite films were synthesized, such as a refractive hybrid optical Ti–O–Si film with 2-phenylphenoxyethyl acrylate [19], a gold-silica nanocomposite [20], a polyurethane foam prepared by a-zirconium phosphate and two biopolymers [21], gel and gold nanorods [22], a fluoropolymer/silica (FP/SiO_2_) organic–inorganic nanocomposite [23], linear colloidal silica nanoparticles [24], polymer nano-alumina nanocomposites [25], raspberry-like structured silica nanocomposites [26], and epoxy resin/silica nanocomposites [27]. Many novel preparation methods were also proposed, e.g., water sol-gel [28], UV-irradiation processes [19], the simple wet impregnation method [29], the organic-inorganic hybrid method [30], a hydrothermal approach [31], simple free radical polymerization [32], the ultraviolet-visible light irradiation process [33], and a novel hot press casting method [34]. In addition, the effects of reaction conditions (e.g., stabilizer concentration, monomer concentration, and solvent composition) on particle morphologies were studied in detail [35].

The organic-inorganic hybrid method is one of the most promising methods for fabricating superhydrophobic surfaces because of its low demand for equipment, simple operation, low cost, large-area fabrication, and easy realization of industrialized production [36]. Compared with other organic materials, fluoride monomers have the advantages of good film-forming characteristics, thermo-stability, photo-stability, transparency, and good mechanical property [37]. Nanoparticles were widely used in many fields because of their excellent performance, considerable material sources, and low prices. Given that most superhydrophobic coatings have weak adhesion strength with substrate surfaces, which restricts their practical applications, the adhesion between the coatings and the substrate must be considered. Combining the outstanding properties of fluorinated acrylate copolymers and nanoparticles, the superhydrophobic coatings obtained by this method could be used for outdoor construction and equipment to protect them from environmental pollution. With development of technology, core-shell structured polymer particles were synthesized. Correspondingly, most of the nanocomposites were prepared by sole nanoparticle, such as Al_2_O_3_, TiO_2_, silica [38,39,40]. Although those nanocomposites mentioned above have excellent performance compared to traditional materials, they also possess a single feature. Along with the progress of technology, those materials endowing a oneness capability could not suffice the need of production and science disquisition. Therefore, a novel composite nanoparticle was presented, such as two kinds of nanomaterials (SiO_2_/TiO_2_) being used to prepare the organic-inorganic nanocomposite emulsion [41].

To our knowledge, although extensive researches focusing on the organic-inorganic nanocomposite have been conducted, the ternary nanomaterials and their properties are rarely investigated. Whether the ternary nanoparticles have the outstanding performance? Accordingly, a novel organic-inorganic hydrophobic nanocomposite film composed of core-shell nanoparticles was prepared by polymerization, in which the inorganic phase was ternary SiO_2_–Al_2_O_3_–TiO_2_ nanoparticles modified by methacryloxy propyl trimethoxyl silane, and the organic phase was made of fluoride and siloxane polymers. Moreover, the properties of the nanocomposite film were studied in detail and presented in this paper.

## 2. Experiments

### 2.1. Raw Materials

Hexafluorobutyl methacrylate (HFMA) and dodecafluoroheptyl methacrylate (DFMA) were supplied by Xuejia company (Harbin, China) of China. Methyl methacrylate (MA), acrylic ester, butyl acrylate, methacrylic acid, tetraethyl orthosilicate (TEOS) butyl titanate [Ti(OBu)_4_], aluminium isopropoxide and hydroxyethyl methylacrylate (HEMA) were provided by Shanghai chemical reagent company in Shanghai, China. Methacryloxy propyl trimethoxyl silane (KH570) was purchased from Wuhan University in China. Ethanol, potassium persulfate, ammonia water (NH_3_·H_2_O, 25% mass of water), OP10 emulsifier (effectual component is alkylphenol ethoxylates) and DNS86 emulsifier (effectual components are acacia and sodium alkyl benzene sulfonate) were obtained from a chemical market. All of the chemicals purchased were reagent grade and used without further purification. Deionized water was used for all preparation and treatment process.

### 2.2. Main Instruments and Characterization

The morphology of the nanoparticles was characterized by field-emission high resolution transmission electron microscope (TEM) of JEM-2100F from JEOL Co. in Tokyo, Japan. The accelerating voltage and the point resolution were of 40 kV and 0.19 nm, respectively. The property of the polymer emulsion film was measured by field emission scanning electron microscope (SEM) of Nova Nano SEM 230 from FEI Electron Optics B.V Co. in the Prague, Czech Republic. X-ray diffractometer (XRD) patterns were collected on an X-ray diffractometer (model of D/Max 2500 from Rigaku in Akishima, Japan) with CuK(α) radiation (λ = 1.5406 Å) at an accelerating voltage of 40 kV and a current of 40 mA. The spectra were collected from 2θ in the range of 5°–80°. The chemical structures of the samples were characterized with spectroscopy technique (FTIR). FTIR spectra were recorded on Nicolet 6700 from Thermo Electron Scientific Instruments Co. in Minneapolis, MN, USA, spectrometer in the region of 350–12,500 cm^−1^. The samples were prepared in the form of pellets with KBr and measured in absorption mode. All specimens were dehydrated by vacuum drying chamber. The adsorption of the emulsifiers on the nanomaterials was characterized by UV spectrophotometry of UV2450 from Shimadzu Co. in Kyoto, Japan. The spectra were collected in the range of 190 nm–900 nm, and the corresponding point resolution was of 0.1 nm. In addition, the dynamic contact angle meter (CA) with the model of HARKE-DWK from Beijing Hake Co. (Beijing, China) was also used to determine the hydrophobic performance of the film.

### 2.3. Experimental Procedures

#### 2.3.1. Preparation Process of Ternary SiO_2_–Al_2_O_3_–TiO_2_ Nanomaterials

In the synthesis process, tetraethyl orthosilicate (TEOS), butyl titanate [Ti(OBu)_4_] and aluminium isopropoxide were used to prepare ternary SiO_2_–Al_2_O_3_–TiO_2_ nanoparticle by a sol-gel method using ethanol (C_2_H_5_OH) as the solvent. The appropriate mole ratio of ethanol, water, organic alkoxide, and ammonia for synthesizing ternary nanomaterials was 60:1:1:0.1, and the organic alkoxide included ethyl silicate (TEOS), butyl titanate [Ti(OBu)_4_], and aluminium isopropoxide (C_9_H_21_AlO_3_) with a molar ratio of 0.85:0.05:0.10. The preparation temperature was 55 °C with continuously stirring at 100 rpm for 24 h. In order to discuss the effect of the components’ amounts on morphology of ternary nanoparticles, different amount of organic alkoxide, alcohol, and ammonia (with a ratio of 2, 75, and 0.1) were designed during preparation.

#### 2.3.2. Adsorption Processes of the Emulsifiers on the Nanomaterials

Prior to synthesizing the organic-inorganic nanocomposite, the ternary nanoparticles should be dissolved into the dissolution by the emulsifier. Therefore, the adaptation between the nanoparticles and emulsifier should be investigated. In the present work, two types of emulsifiers, namely, OP10 and DNS86, were selected. Then, the effects of the characteristics of the nanomaterials on the adsorption of the surfactant were studied. The concentrations of the OP10 and DNS86 were 0.2% and 0.05%, respectively. During the measurement, the solution was dispersed by ultrasonic dispersion for 2 min and stilled for 20 min. High speed centrifugation was explored to separate the silica nanoparticles with 10,000 r/min for 20 min. The supernatant was taken out and tested by the ultraviolet spectrophotometer of UV2450 from Shimadzu Co. in Kyoto, Japan. The characteristic waves of the OP10 and DNS86 were 244 nm and 223 nm, respectively.

#### 2.3.3. Preparation of Organic-Inorganic Hydrophobic Nanocomposite Emulsion

According to the emulsion polymerization, there were three steps for synthesizing organic-inorganic hydrophobic nanocomposite emulsion. First, the seed emulsion was prepared. The amount of nanomaterials, water, surfactant (KH570), and sodium bicarbonate (NaHCO_3_ solution with pH = 6.5) introduced into a flask with a ratio of 0.5:80:0.1:0.2, and the mixture was stirred for 1 h at 30 °C. Then the temperature of the system was heated up to 70 °C, and the mixture composed of methyl methacrylate, styrene, surfactant, and water with a ratio of 2.5:2.5:3.2:0.08 was added into the flask. Meanwhile, the initiator (i.e., potassium persulfate solution) was added as well. The reaction continued for 2 h, and then the prepolymer was synthesized. The temperature was heated up to 75 °C, and the amount of methyl methacrylate, styrene, butyl acrylate, methacrylic acid, HEMA, water, and surfactant with a ratio of 2.4:1.6:3.3:0.4:0.6:8.4:0.21 was subsequently poured into the flask. At the same time, the amount of the initiator (i.e., potassium persulfate solution) added into flask was 0.12% of total monomers, as well. The reaction continued for 2 h. Eventually, the organic-inorganic hydrophobic nanocomposite emulsion was prepared. In consequence, the synthesis system was kept at 75 °C, and the mixture of butyl acrylate, hexafluorobutyl methacrylate (HFMA), dodecafluoroheptyl methacrylate (DFMA), KH570, water, surfactant, and initiator with a ratio of 1.5:4:2:1:8.5:0.26:0.1 was introduced into the flask. The mixture solution reacted continuously for 6 h at 75 °C and was stirred at 100 rpm. After being cooled to room temperature, the pH value of the solution was adjusted to 7, and the organic-inorganic core-shell nanocomposite emulsion was obtained. The methacryloxy propyl trimethoxyl silane (KH570) was used as the surface medication agent. The OP10 and DNS86 were mixed with a mass ratio of 1:1, which was used as the emulsifier. In addition, the whole preparation process was performed under a nitrogen atmosphere.

## 3. Results and Discussion

### 3.1. Synthesis of Ternary SiO_2_–Al_2_O_3_–TiO_2_ Nanomaterials

**Figure 1:**
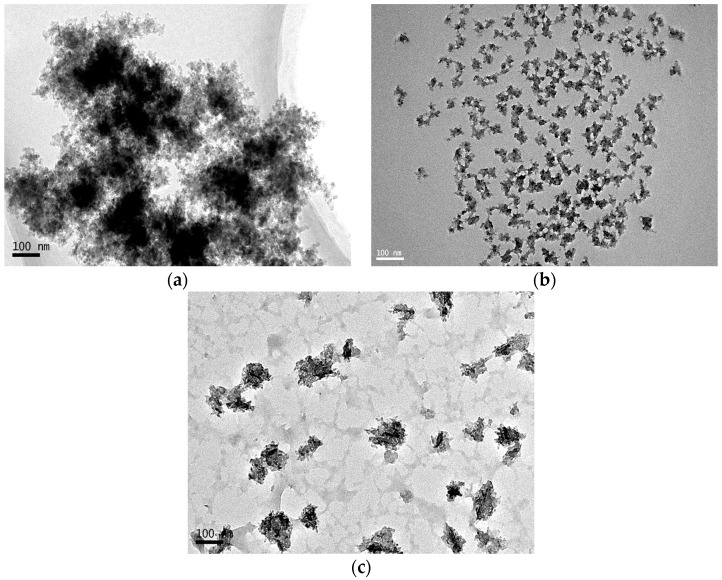
TEM images of ternary nanomaterials. (**a**) More organic alkoxide (with a ratio of 2); (**b**) more alcohol (with a ratio of 75); and (**c**) an appropriate amount of ammonia (with a ratio of 0.1).

According to the procedure in Section 2.3.1, the ternary SiO_2_–Al_2_O_3_–TiO_2_ nanomaterial was prepared, and the TEM spectra were shown in Figure 1.

Figure 1 indicates that the influences of different synthetic parameters on the characteristics of the nanomaterials are obvious. Adding more organic alkoxide (with a ratio of 2) to the system, there is more gel, as shown in Figure 1a, which is due to the hydroxy produced by the hydrolysis reaction as the organic alkoxide reacts with each other. Subsequently, the dehydration synthesis reaction proceeds. The formed productions overlap with each other and produce the network structure. Thus, the apparent viscosity of the system increases and results in the formation of a substantial amount of gel. Under the condition of an appropriate amount of ammonia (with a ratio of 0.1), the synthesis system can produce nanoparticles with a size of approximately 100 nm, as shown in Figure 1c. Meanwhile, if there is more alcohol (with a ratio of 75) in the solution, which will dilute the concentration of organic alkoxide, giving rise to small sized nanoparticles as shown in Figure 1b. As discussed above, a conclusion can be made that the size of nanoparticles is significantly affected by the dose of the reactant and solvent.

### 3.2. Modification of the Ternary Nanomaterials

**Figure 2:**
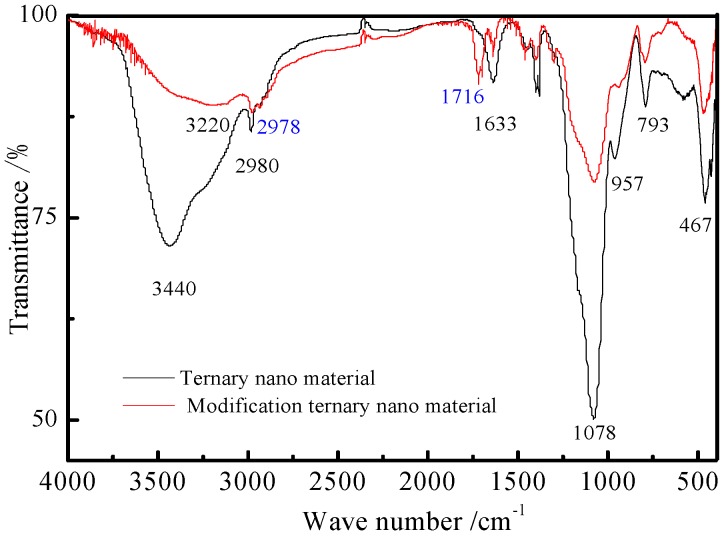
FTIR spectra of the ternary nanomaterials.

Since the nanoparticles cannot directly react with the organic monomers, such as HFMA, DFMA, and so on, the surface modification of the nanomaterials should be conducted. The FTIR analysis for the ternary nanomaterials before and after the surface modification was carried out, and the spectra are shown in Figure 2.

Figure 2 indicates that there are significant differences of the surface characteristics of the nanoparticles before and after surface modification. In Figure 2, the peak at 1100 cm^−1^ can be attributed to the stretching vibrational adsorption of Si–O–Si bond. The peak at 467 cm^−1^ is the bending vibration adsorption peak of Si–O–Si, and the peak at 957 cm^−1^ is the bending vibration adsorption peak of Si–O–H. In addition, the stretching vibrational peak at 2980 cm^−1^ belongs to the group of O–CH, attributed to the chemical structures of the TEOS. The corresponding peak is very weak, which suggests that the TEOS basically reacted. In general, the peak at 3440 cm^−1^ is the adsorption peak of the group O–H and Si–OH, and the peak at 1633 cm^−1^ is the bending vibration adsorption peak of H_2_O. Figure 2 also reveals that the peak at 3440 cm^−1^ corresponding to the stretching vibrational adsorption of -OH bond turns out to be weaker, which indicates that the amount of the hydroxy on the nanomaterial surface decreases. Thus, the water resistance of the silica nanoparticles after surface modification is enhanced. The peak at 2980 cm^−1^ is the adsorption peak of –CH_3_, and the peak at 1716 cm^−1^ is the characteristic peak of C=O. From above analysis, it can be seen that there exists a chemical reaction between the nanomaterials and KH570. Moreover, the peaks at 1000 cm^−1^ and 800 cm^−1^ respectively shift to 1078 cm^−1^ and 793 cm^−1^, which may originate from the vibration absorption peak of Si–O–Al/Ti bond [16].

### 3.3. Adsorption of the Nanomaterials

**Figure 3:**
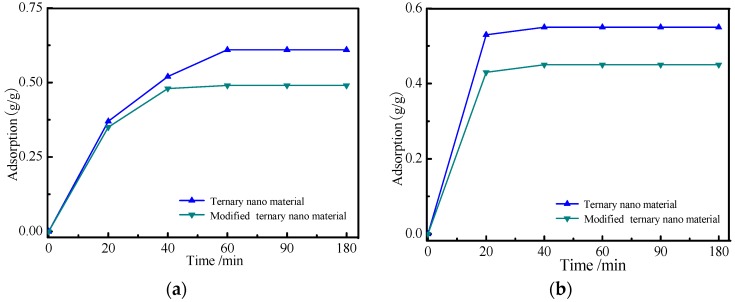
Adsorption curves of nanomaterials against emulsifiers. (**a**) OP10; and (**b**) DNS86.

In order to characterize of the nanomaterials’ surface adsorption, two types of emulsifiers, namely, OP10 and DNS86, were selected to conduct the adsorption process according to Section 2.3.2. The adsorption curves of nanomaterials against emulsifiers were shown in Figure 3.

Figure 3 shows the adsorption dosage and rate of nanoparticles were different towards different kinds of emulsifiers (surfactants). Using a modified ternary nanomaterial as an example, the adsorption dosage of OP10 is greater, the adsorption rate of DNS86 is quicker. This is because OP10 is considered as a non-ionic surfactant, which is absorbed on the nanomaterials’ surface by the way of physisorption. However, DNS86 is a form of ionic surfactant, and the mechanism of the adsorption process is the charge adsorption. In comparison to the nanomaterials, the dosage of the surfactant absorbed by surface modified nanomaterials decreases, which is because the nanomaterial surface absorbs sufficient KH570 and results in a decrease of the exposed area. In Figure 3, it also indicates the type of emulsifier infused into solution should be based on the nanoparticles’ species and dosage during the synthesis process.

### 3.4. Synthesis of Organic-Inorganic Hydrophobic Nanocomposite Emulsion

**Figure 4:**
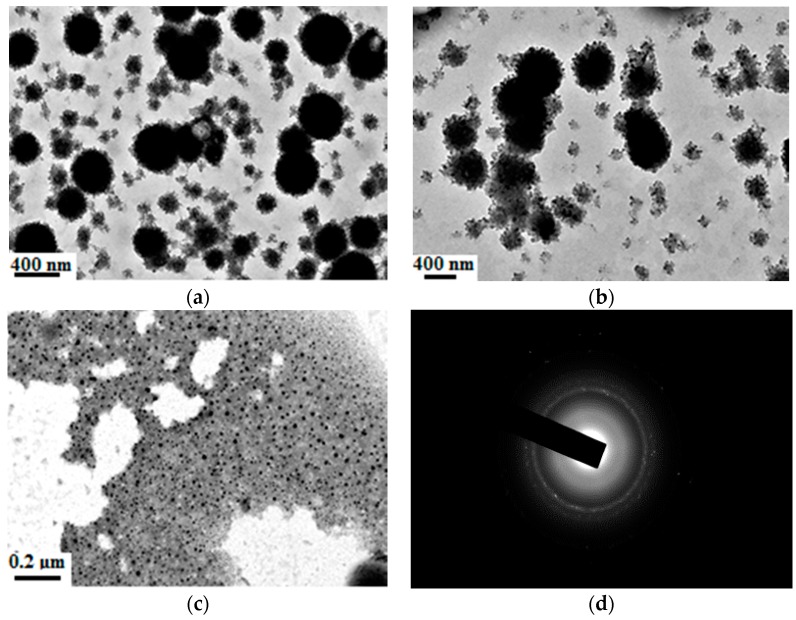
TEM spectra of organic-inorganic super hydrophobic nanocomposite emulsion. (**a**) Strawberry particle; (**b**) small particles adsorbed on the big particles; (**c**) film; and (**d**) diffraction pattern.

According to the Section 2.3.3, the organic-inorganic hydrophobic nanocomposite emulsion was prepared. To study the morphology characteristics of the organic-inorganic core-shell nanocomposite emulsion, the TEM analysis was made, as shown in Figure 4.

Figure 4a shows that the morphology of the organic-inorganic core-shell nanocomposite particles were in the form of strawberry core-shell structure, in which the inorganic phase was ternary SiO_2_–Al_2_O_3_–TiO_2_ nanoparticles modified by KH570, and the organic phase was made of fluoride and siloxane polymers. Meanwhile, there were many small particles scattered in the system or adsorbed on the polymer particles’ surface, as shown in Figure 4b. The reason for this maybe the polymer monomers added into system reacts with each other and produces small particle before reacting with the nanocomposite particle. A crystal of the film is analysed on the field-emission high-resolution transmission electron microscope as shown in Figure 4c,d. The diffraction pattern reveals that the nanomaterial is of a crystalline structure, which is caused by the high temperature and the synthetic environment leading to the unit cell transformation of nanomaterials.

### 3.5. Effect of Temperature on the Nanocomposite Particle

**Figure 5:**
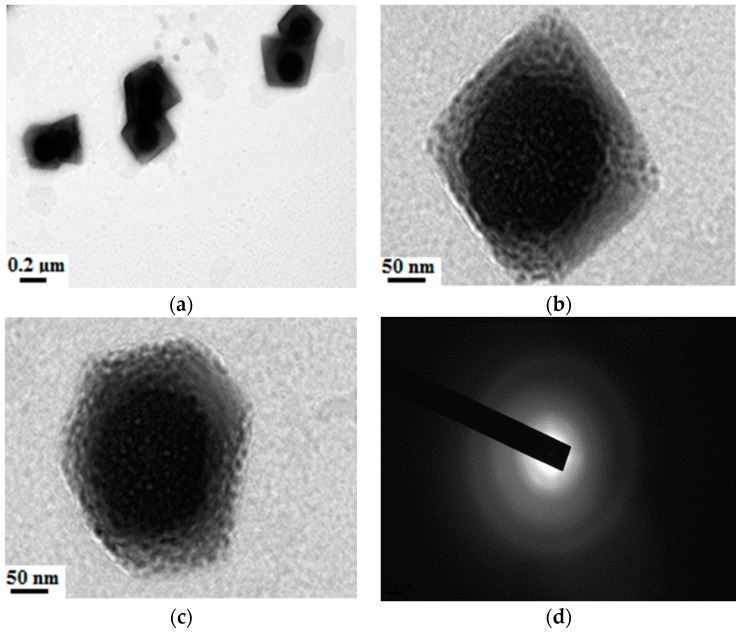
TEM spectra of organic-inorganic super hydrophobic nanocomposite emulsion at 80 °C. (**a**) Tetrahedral particle; (**b**) cube particle; (**c**) octahedral particle; and (**d**) diffraction pattern.

Based on the results aforementioned, the synthesis temperature was further increased up to 80 °C and the morphology characteristics of the nanoparticles were determined, as shown in Figure 5.

Figure 5 indicates that the effect of the temperature on the morphology of the organic-inorganic hydrophobic nanocomposite emulsion is significant, which is in the form of polyhedron. This is because the temperature affects the reaction process and changes the unit cell structure of the nanoparticle. By analysis of the TEM diffraction pattern, the results show that the diffraction pattern disappears, as shown in Figure 5d, which reveals the amorphous structure of the nanomaterials. In comparison with the morphology of the organic-inorganic hydrophobic nanocomposite in Figure 4 and Figure 5, it can be seen the shape of the particles changes from spherical to polyhedron. This may be due to the organic polymer around the nanocomposite sphere softening under high temperature (i.e., 80 °C), and the particles stack and extrude each other. Thus, the shape of the nanoparticle turns into a polyhedron with the decreasing of temperature. This polyhedron nanocomposite was also reported by [42].

### 3.6. Effect of MA and HFMA on the Nanocomposite Particle

**Figure 6:**
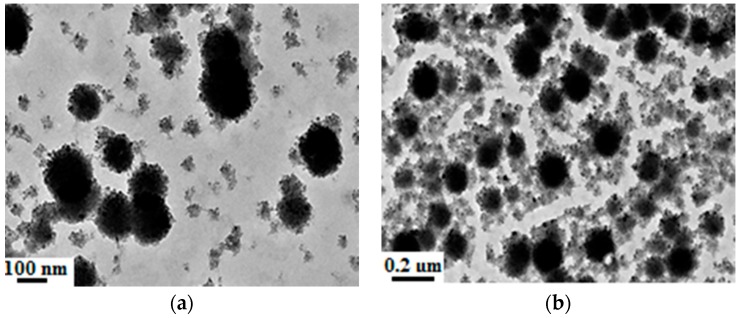
TEM images of nanocomposite particle synthesized with more MA and HFMA. (**a**) MA (with a ratio of 3.0); and (**b**) HFMA (with a ratio of 1.0).

Since the MA and HFMA were the most important components in the nanocomposite particles, this study investigated the effect of MA and HFMA dosage on the morphology of the nanoparticles. Based on Section 2.3.3, the normal amount of methyl methacrylate, styrene, butyl acrylate, methacrylic acid, HEMA, water, and surfactant with a ratio of 2.4:1.6:3.3:0.4:0.6:8.4:0.21 was adopted during the synthesis process. In order to investigate the effect of more MA and HEMA on the morphology of nanocomposite particle, the ratios of MA and HEMA were increased (i.e., 3.0 and 1.0, respectively). Figure 6 shows the TEM spectra of nanocomposite particle synthesized with more MA and HFMA.

Figure 6 indicates that the morphology of the obtained composite particles were changed when more MA and HFMA (i.e., the ratio of 3.0 and 1.0, respectively) were introduced into the same experiment performed above, forming irregular core-shell (snowman-like) morphologies for MA, and irregular core-shell (strawberry-like) morphologies for HFMA. The KH570 with a strong affinity for the nanoparticle surface hydrolyzes rapidly to form silanol. On the one hand, the molecule of KH570 bonded with bare nanoparticle surface by –O–Si– chemical bond; on the other hand, it also reacted with MA and HFMA monomers and generated high-molecular polymer. Eventually, the organic polymers were adopted on the bare nanoparticle surface. MA, as a component, can react with the unsaturated bonds of KH570 to modify the bare nanoparticles. With the elongation of reaction time, more and more MA reacted to form a shell outside the nanoparticle. At last, the nanoparticles were all covered by the polymer of MA, as shown in Figure 6a. Simultaneously, because more HFMA was added into the synthesis system, more and more polymer particles were generated without the shell structure. This was due to partial HFMA lacking enough time to react with KH570 on the nanoparticles’ surface, so the HFMA reacted with each other and produced the polymer particles without the shell structure. Eventually, there were more and smaller polymer particles generated after a long reaction time. In comparison with MA, the effect of HFMA on the nanocomposite particles was different, which generated the outside shell structure of the polymer nanoparticles.

### 3.7. XRD Spectra of the Nanocomposite Film

**Figure 7:**
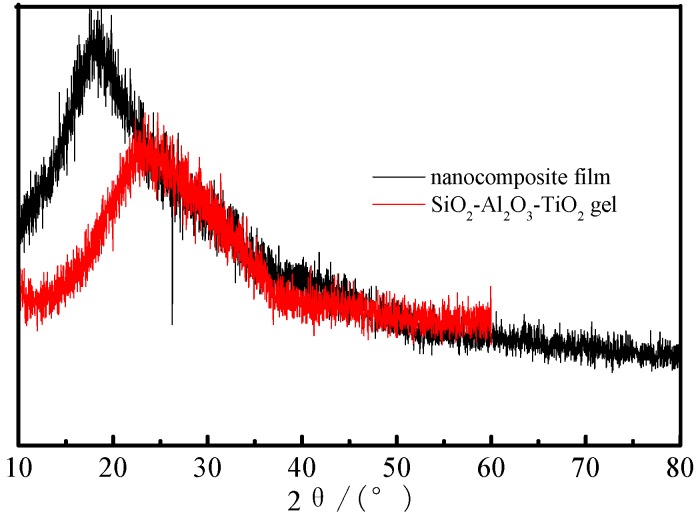
XRD spectra of the specimens.

In order to discuss the phase and characteristics of the organic-inorganic hydrophobic nanocomposite with a ternary composition SiO_2_–Al_2_O_3_–TiO_2_, the dried samples were firstly ground into powder and then used for XRD measurements. The corresponding XRD spectra of the specimens are shown in Figure 7.

Figure 7 shows that there is a wide peak of the organic-inorganic hydrophobic nanocomposite with ternary SiO_2_–Al_2_O_3_–TiO_2_ as the core, which reveals that the polymer is amorphous. Compared with the spectrum of the ternary SiO_2_–Al_2_O_3_–TiO_2_ gel, it can be seen that the peak of the nanocomposite film becomes stronger and the angle of the peak shifts to the low angle direction, which might be due to the difference of polymers.

### 3.8. Hydrophobicity of the Nanocomposite Film

**Figure 8:**
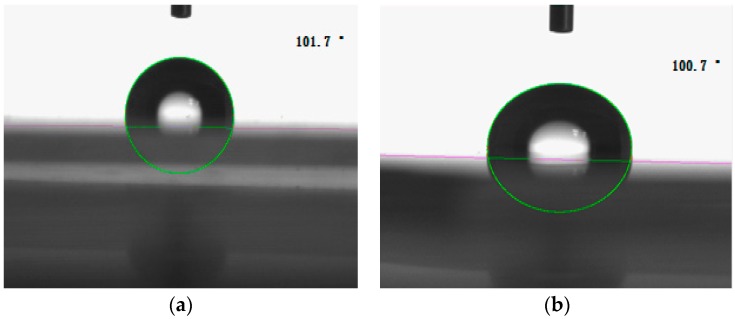
Hydrophobicity of the nanocompostie film. (**a**) Ternary SiO_2_–Al_2_O_3_–TiO_2_ nanoparticles; and (**b**) SiO_2_ nanoparticles.

The hydrophobicity of the novel organic-inorganic hydrophobic nanocomposite film composed of SiO_2_–Al_2_O_3_–TiO_2_ and SiO_2_ nanoparticles was tested by the dynamic contact angle meter (CA), and the corresponding results are shown in Figure 8.

According to Figure 8a, the results show that the contact angle of the novel organic-inorganic hydrophobic nanocomposite film composed of SiO_2_–Al_2_O_3_–TiO_2_ was greater than 101.7°, which reveals that the organic-inorganic nanocomposite film composed of ternary SiO_2_–Al_2_O_3_–TiO_2_ nanoparticles is hydrophobic. This is because the surface of the core-shell structure of the particle is made of fluoride and siloxane polymers. In addition, it can also be seen from Figure 8b that the contact angle of the organic-inorganic hydrophobic nanocomposite film composed of SiO_2_ was 100.7°. Therefore, the organic-inorganic core-shell nanocomposite film has an excellent hydrophobicity.

### 3.9. SEM Analysis of the Nanocomposite Film

**Figure 9:**
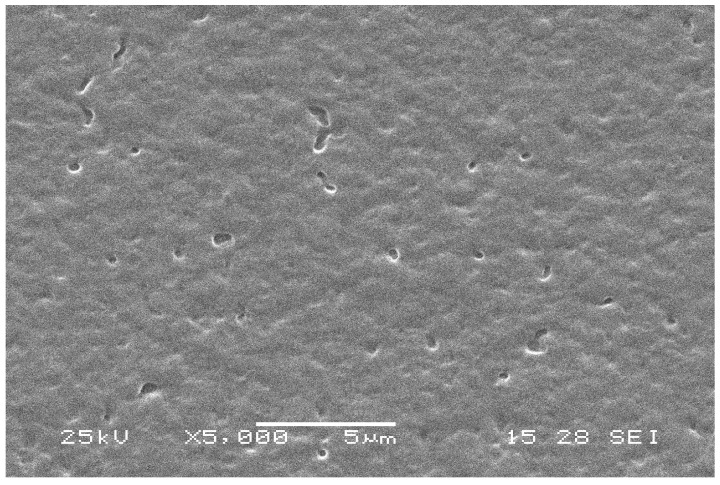
SEM image of the film with SiO_2_–Al_2_O_3_–TiO_2_ nanoparticles as the core.

In order to compare the difference of the nanocomposite with different core compositions of silica and ternary SiO_2_–Al_2_O_3_–TiO_2_, the specimens were characterized by the SEM analysis, and the corresponding SEM image was shown in Figure 9.

From Figure 9, it can be seen that the nanocomposite film with ternary SiO_2_–Al_2_O_3_–TiO_2_ nanoparticles as the core are very rough with uneven areas, and there exists a coarse surface. This may be due to many tiny spheres (seen in Figure 6) solidifying around the large nanocomposite particles’ surface, when the nanocomposite particle film was prepared. Moreover, numerous tiny holes are present on the surface of the film, which may be caused by the space among the nanoparticles unfilled by tiny particles. It was pointed out that these rough areas and the small holes would increase the hydrophobicity of the film [24]. To sum up, the surface morphology of the film with SiO_2_–Al_2_O_3_–TiO_2_ nanoparticles as the core could be demonstrated by SEM analysis.

## 4. Conclusions

The morphology of the organic-inorganic core-shell nanocomposite particles were in the form of a strawberry core-shell structure, in which the inorganic phase were ternary SiO_2_–Al_2_O_3_–TiO_2_ nanoparticles modified by KH570, and the organic phase was made of fluoride and siloxane polymers. Meanwhile, there were many small particles scattered in the system or adsorbed on the surface of the polymer particles. The temperature can affect the morphology and the crystalline structure of the polymer film. At high temperatures, the nanomaterials will be amorphous in structure. The contact angle of the nanocomposite polymer film with the ternary SiO_2_–Al_2_O_3_–TiO_2_ cores was greater than 101.7°, which reveals that the novel organic-inorganic polymer nanocomposite film has an excellent hydrophobicity. SEM analysis shows that the surface of nanocomposite film is rough with numerous small holes.
